# Elevated defeatist performance beliefs predict state increases in negative symptoms in daily life in clinical high-risk for psychosis youth: implications for mobile health treatments

**DOI:** 10.1007/s00406-025-02143-4

**Published:** 2025-11-10

**Authors:** Lauren Luther, Ian M. Raugh, Luca Bela G. Webber, Paul M. Grant, Aaron T. Beck, Vijay A. Mittal, Elaine Walker, Gregory P. Strauss

**Affiliations:** 1 Department of Psychology, University of Alabama at Birmingham, Birmingham, AL USA; 2 Department of Psychiatry, Douglas Mental Health Institute, McGill University, Montréal, Québec Canada; 3 Center for Recovery-Oriented Cognitive Therapy, Beck Institute, Bala Cynwyd, PA USA; 4 Department of Psychiatry, University of Pennsylvania, Philadelphia, PA USA; 5 Department of Psychology, Northwestern University, Evanston, IL USA; 6 Institutes for Policy Research (IPR) and Innovations in Developmental Sciences (DevSci), Psychiatry, Medical Social Sciences, Northwestern University, Evanston, IL USA; 7 Department of Psychiatry and Behavioral Sciences, Emory University, Atlanta, GA USA; 8 Department of Psychology, Emory University, Atlanta, GA USA; 9 Department of Psychology, University of Georgia, 125 Baldwin St, Athens, GA 30602 USA

**Keywords:** Cognitive behavioral therapy, Clinical high-risk, Psychosis, Ecological momentary assessment, Psychosis

## Abstract

**Background:**

Negative symptoms are a strong predictor of conversion to a formal psychotic disorder in youth at clinical high-risk for developing psychosis (CHR). Identification of temporally precise mechanisms underlying increases in negative symptoms could enhance early intervention and specifically support the utility of mobile health treatments. Guided by Cognitive Behavioral models of psychopathology, we examine whether a core type of biased thinking—defeatist performance beliefs (DPB)—is a real-world mechanism of negative symptoms as well as a secondary symptom that is common in CHR youth: depressed mood.

**Methods:**

CHR youth (*n* = 119) and healthy control (CN; 59) subjects completed ecological momentary assessment surveys assessing DPB, negative symptoms, and depressed mood for six days.

**Results:**

CHR youth reported elevated DPB in daily life compared to CN. Greater DPB were associated with greater concurrent negative symptoms and depressed mood in daily life. Time-lagged analyses demonstrated that increased DPB at time *t* led to elevations in negative symptoms and depressed mood at *t* + 1 above and beyond the effects of the respective symptom at time *t;* DPB also varied across time of day, study day, day of the week, activity context, and social partners.

**Conclusions:**

DPB may be a promising shared mechanism contributing to negative symptoms and depressed mood in CHR youth in their daily life. Findings also provide proof-of-concept support for the utility of mobile health treatments targeting DPB by identifying key moments where DPB fluctuate in CHR youths’ everyday environments.

**Supplementary Information:**

The online version contains supplementary material available at https://doi.org/10.1007/s00406-025-02143-4.

## Introduction

Schizophrenia (SZ) is associated with extremely high rates of functional disability and health care costs. One of the primary contributors to this functional disability is negative symptoms. These symptoms typically develop in the prodromal (i.e., pre-illness) period prior to attenuated positive symptoms [1], indicating that negative symptoms are one of the earliest indicators of psychosis risk. The severity of negative symptoms is also a robust predictor of conversion to a formal psychotic disorder [2–5]. In addition, given negative symptoms' detrimental impact on social and role functioning [6–8], these symptoms are also one of the primary reasons why youth in the prodromal period initially enter treatment [9]. However, our ability to effectively prevent or even treat these symptoms in the prodromal period is hampered by a limited understanding of the critical mechanisms underlying negative symptoms during this period, including in youth at clinical-high risk for psychosis (CHR) or those who are experiencing attenuated psychotic symptoms and declines in functioning. Identification of mechanisms vital to negative symptoms in CHR youth could isolate critical treatment targets that may offset the severity or development of negative symptoms and associated functional disability in CHR youth.

One model of negative symptoms that has demonstrated promise for elucidating impactful mechanisms of negative symptoms in those with a formal psychotic disorder is the Cognitive Behavioral Therapy (CBT) model of negative symptoms [10]. This model posits that negative thinking patterns around capability and social value are a fundamental cause of the development and maintenance of anhedonia, avolition, and asociality [10, 11] These negative thinking patterns are theorized to develop prior to the onset of a formal psychotic disorder, arising from a mixture of challenging life experiences (difficulties at school, challenging personal relationships, unmet needs, etc.) and vulnerability for a psychotic disorder that predisposes a person to be more likely to have unhelpful schemas that bias information processing to yield a negative belief system [10].

An increasing body of empirical work has demonstrated support for this model by examining a specific type of biased thinking that is central to the CBT model of negative symptoms—defeatist performance beliefs (DPB), which are beliefs about the role of potential failure, that are extremely personally costly [10, 11]. In SZ, DPB have been found to be consistently elevated relative to healthy controls (CN) [11–16]. These DPB elevations in turn are significantly associated with greater negative symptom severity [17]. These findings have formed the basis of Recovery-Oriented Cognitive Therapy (CT-R) [18] and Cognitive Behavioral Social Skills Training (CBSST; , which have demonstrated efficacy in improving negative symptoms through targeting DPB in SZ [18–20]. In addition, there is also increasing evidence that DPB are elevated in at-risk groups compared to CN, including individuals with schizotypy [21] and CHR youth [12, 22, 23]. CHR youth who demonstrate greater DPB have also been found to demonstrate increased negative symptom severity [12, 22–24]. Recent evidence suggests that DPB severity is highly similar between CHR and SZ [22], supporting the model’s assertions that these beliefs develop and are formed prior to the development of a full-blown psychotic disorder. However, a limitation of this body of work is that it derived from laboratory-based trait-based measurements of DPB and negative symptoms that have limited ecological validity. To address this, we recently used Ecological Momentary Assessment (EMA) to assess DPB and negative symptoms in daily life in SZ and CN [25]. Mirroring the trait DPB findings, SZ reported significantly greater state DPB elevations in daily life compared to CN. Further, we also isolated a putative causal pathway, identifying that greater state DPB at time *t* predicted subsequent increases in negative symptoms at time *t* + 1. While these findings support the ecological validity of the model in those who already have a formal psychotic disorder diagnosis, a key part of the model that has yet to be tested relates to the state effect of DPB elevations contributing to subsequent negative symptoms in daily life in CHR youth. Testing this in CHR youth and establishing a temporally precise link between DPB and prospective negative symptoms in daily life is important for elucidating the viability and utility of targeting DPB in early intervention and prevention efforts. In addition, there is also limited empirical understanding of the time-course of DPB in daily life in CHR, including how different environmental contexts may differentially influence DPB severity. Answering these questions will be critical for optimizing a CBT digital therapeutic for negative symptoms, including identifying when, where, and how to deliver intervention content to yield the greatest effects on DPB and negative symptoms.

The current study aimed to address these gaps using Ecological Momentary Assessment (EMA) to study state models of how daily fluctuations in DPB predict changes in negative symptoms in CHR youth and CN. A secondary aim was to clarify the role of DPB on depressed mood in daily life. We focused on depressed mood for several reasons. First, studies have found that depressed or low mood is a feature of depression symptoms that is distinct from negative symptoms [26]. Second, there is high frequency of depressed mood in CHR youth, and many CHR youth go on to develop mood disorders rather than formal psychotic disorders [27–29], Third, similar to negative symptoms, CBT models posit a role of defeatist beliefs in the development and maintenance of depressed mood. Finally, identification of whether DBP might be a shared mechanism of both depressed mood and negative symptoms in CHR youth could help to isolate treatment targets that have the greatest potential impact. Based on our prior study examining state DPB in SZ and prior DPB evidence with the DPB trait scale in CHR [12, 22, 23, 25], we made the following hypotheses: (1) CHR youth would demonstrate increased DPB via EMA compared to CN; (2) DPB measured via EMA would predict concurrent levels of EMA-assessed negative symptoms and depressed mood; (3) DPB at time *t* would predict increases in negative symptoms and depressed mood at time *t* + 1. In addition, to inform whether digital therapeutics may have utility in targeting DPB to improve symptoms, we also conducted several additional analyses aimed at identifying when and how DPB fluctuates across time and context (i.e., to identify if there are central times when DPB should be targeted).

## Methods

### Participants

Participants included 59 healthy controls (CN; 1,941 EMA samples) and 119 CHR youth (3,334 EMA samples). CHR participants were recruited across 3 research programs: (1) the Georgia Psychiatric Risk Evaluation Program (G-PREP) in Athens, GA, (2) the Northwestern University Adolescent Development and Preventative Treatment (ADAPT) research program in Evanston, IL, and (3) the Mental Health and Development (MHAD) Program at Emory University in Atlanta, GA. These programs specialize in conducting evaluations for youth with early psychosis symptoms. Written informed consent for a protocol with IRB approval from the local institution was provided by all participants. The study was performed in accordance with the ethical standards laid down in the 1964 Declaration of Helsinki and its later amendments.

Youth at CHR met criteria for a psychosis-risk syndrome on the Structured Interview for Psychosis-Risk Syndromes (SIPS; Miller et al., 2003). Of the 119 CHR participants, 116 met criteria for the Attenuated Positive Symptoms Syndrome (APSS; i.e., rating of 3–5 on at least one SIPS positive symptom item). Of these, 65 met criteria for progression, 49 for persistence, and 2 for partial remission. The remaining CHR participants met criteria for multiple syndromes: APSS Persistence and BIPS full remission (*n* = 1); APSS persistence, BIPS full remission, and GRD full remission (*n* = 1); APSS progression and GRD progression (*n* = 1). CHR participants did not meet lifetime criteria for a psychotic disorder via the Structured Clinical Interview for DSM-5 (SCID-5; First et al., 2015).

CN recruitment was facilitated by using printed and online advertisements in the local community. CN participants were eligible if they did not meet SCID-5 criteria for a current psychiatric disorder, have a lifetime history of a psychotic disorder, or have a family history of a psychotic disorder. Participants in both groups reported no lifetime history of neurological disease. CHR and CN participants did not significantly differ in age, personal education, parental education, sex, or race. However, CHR completed fewer EMA surveys than CN (see Table 1).

**Table 1:** Demographic and clinical characteristics

Variable	CHR (*n* = 119)	CN (*n* = 59)	Test Statistic	*p*
Age; M (SD)	22.34 (3.99)	21.47 (3.07)	*t* = -1.47	0.14
Male; *n* (%)	27 (22.7%)	14 (23.7%)	χ^2^ = 0.02	0.88
Personal education	13.89 (2.70)	14.50 (1.49)	*t* = 1.62	0.11
Parental education	15.34 (2.97)	15.85 (2.83)	*t* = 1.10	0.27
Race	n (%)	n (%)	χ^2^ = 4.17	0.53
African American	16 (13.4%)	5 (8.5%)		
Asian American	16 (13.4%)	7 (11.9%)		
Caucasian	62 (52.1%)	39 (66.1%)		
Biracial	12 (10.1%)	3 (5.1%)		
Hispanic/ Latino	12 (10.1%)	4 (6.8%)		
Other	1 (0.8%)	1 (1.7%)		
Survey Adherence	57.83%	71.19%	*t* = 3.49	< 0.001

Adherence is the percentage of eight available surveys completed per day

*CHR* = clinical-high risk for psychosis group, *CN* = Control group

### Procedures

All components of the study were completed over the course of one week. Procedures followed those reported in our past CHR and SZ studies [25, 30, 31]. Procedures specific to the current study are detailed below.

*Initial Laboratory Visit*. After informed consent was provided, participants completed diagnostic and symptom interviews. All participants were rated on the SCID-5, and CHR participants were also rated on the SIPS. Once diagnostic consensus was reached, participants were then trained on the mobile application used for EMA surveys. Training included downloading the mEMA app (ilumivu.com) onto their personal smartphone, demonstrating how to use the app, and completing a practice EMA survey.

*EMA Surveys.* Participants received EMA surveys through the mEMA app for 6 days. Participants were notified at 8 randomized times within 90-minute time periods between 9AM to 9PM of the availability of surveys. Surveys incorporated skip logic and were designed to take less than 5 min to complete to reduce participant burden. Each EMA survey included items assessing DPB, negative symptoms, and mood (see supplement for full items). EMA DPB items were used previously in SZ [25] and modified from the trait DPB scale through discussions with seminal authors of the DPB trait scale used with SZ (AT Beck and PM Grant). Three items most central to the DPB construct were selected: “I have to do well all the time or people will not respect me.”, “If you cannot do something well, there is little point in doing it.”, and “If I fail at all, it is as bad as being a complete failure.” Participants were asked to rate the EMA DPB items as follows: “How much do you agree or disagree with the following right now.”

Each EMA survey also contained items assessing the negative symptom domains of anhedonia, avolition, and asociality. These items were modeled after the BNSS and prior EMA studies [32, 33] and assessed both the internal and behavioral components of these symptom domains. The behavioral component was assessed via checklists indicating engagement in social (asociality), recreational (anhedonia), or goal-directed (avolition) activities. Internal experience was then assessed via questions querying the level of enjoyment (consummatory and anticipatory) for endorsed recreational and social activities for anhedonia, level of interest in goal-directed behaviors for avolition, and level of interest for social interaction for asociality. Depressed mood was indexed on each EMA survey through a question asking participants to rate how sad, downhearted, or unhappy they were right now. Finally, EMA survey items also captured current activity, location, social partner (if applicable), and method of social interaction. Survey items are in the supplement.

*Return to Laboratory.* After the EMA period, participants were compensated $1 for each EMA survey completed and received a bonus of up to $60 if they completed 5 or more surveys per day and > 80% of the surveys. Participants also received $30 per hour of interviews.

### Data analysis

To assess whether DPB levels differed in daily life between CHR and CN, we used a multi-level mixed-effects model with random intercepts per person per day. Concurrent associations between DPB and negative symptoms or depressed mood were examined with mixed-effects models with negative symptom domains (anhedonia, avolition, or asociality) or depressed mood serving as the dependent variables and DPB, group (CHR, CN), and the interaction between DPB and group as the independent variables . Time-lagged associations were examined with autoregressive models where the symptom of interest at *t* + 1 regressed onto DPB, Group, and the interaction thereof while controlling for the symptom of interest at *t*. DPB changes across the EMA period were also evaluated using mixed-effects models with random intercepts per person per day. In these models, DPB was the dependent variable predicted by the time factor of interest (time of day, study day, day of week), group (CHR, CN), and the interaction between the predictor of interest and group. Context effects were evaluated using mixed effects models with random intercepts per person per day. Context models measured the effects on DPB of group (CHR, CN), context (current activity, location, social partner, or social modality), and a group X context interaction. See [30, 34] for more details. Models contained unstructured covariance structures with random slopes for the primary predictor (DPB or time factor) and random intercepts per person per day; however, models were simplified as needed to avoid singular fit (e.g., removing random slopes, then random intercepts).

## Results

*Group differences.* Compared to CN, the CHR group reported significantly greater DPB via EMA (p < .001).

### Symptom associations

*Concurrent effects.* Increased DPB was associated with greater levels of concurrent negative symptoms in all negative symptom domains (anhedonia, asociality, avolition) and depressed mood (see Table 2). The Group X DPB interactions were not significant for all negative symptom domains or depressed mood. This suggests that the strength of the association between DPB and both negative symptoms and depressed mood was similar in both CHR and CN. However, there was a main effect of group for depressed mood, with depressed mood being significantly higher in CHR than CN.

**Table 2:** Summary of concurrent models

			Model terms								
			DPB			Group			DPB X Group		
Outcome	R^2^ total	R^2^ fixed effect	Beta	*t*	*p*	Beta	*t*	*p*	Beta	*t*	*p*
Anhedonia	0.31	0.04	0.19	5.49	< 0.001	− 0.14	-1.85	0.06	− 0.11	-1.55	0.12
Asociality	0.33	0.01	0.07	2.02	0.04	− 0.03	− 0.31	0.75	− 0.09	-1.17	0.24
Avolition	0.26	0.02	0.15	4.51	< 0.001	− 0.12	-1.51	0.13	− 0.11	-1.59	0.11
Depressed mood	0.42	0.17	0.37	9.18	< 0.001	− 0.36	-4.16	< 0.001	− 0.13	-1.61	0.11

Standardized betas are reported

*DPB* = defeatist performance beliefs

*Autoregressive effects.* Autoregressive models (see Table 3) demonstrated significant main effects for DPB in the anhedonia, avolition, and depressed mood models but not asociality (*p* = .0.06). There were significant main effects of each negative symptom domain and depressed mood at time *t* on the negative symptom domain in the model measured at *t* + 1. There was also a significant Group X DPB interaction for all three negative symptom domains but not depressed mood. In all three negative symptom models, in the CHR group, there was a positive association in the CHR group, such that increased DPB at time *t* was associated with greater negative symptoms for all domains (anhedonia, avolition, and asociality) at *t* + 1 after controlling for the negative symptom domain in the model at time *t*. On the other hand, the association in the CN group was negligible or slightly negative. In the depressed mood model, there was also a significant main effect of group, with CHR reporting higher depressed mood than CN. Thus, the detrimental effect of DPB on prospective negative symptoms is stronger in CHR than CN, while the effect of DPB on prospective depressed mood is similar across CHR and CN.

**Table 3:** Summary of autoregressive models

			Model terms											
			DPB			Group			DPB X group			AR (Time 1)		
Outcome	R^2^ total	R^2^ fixed effects	Beta	*t*	*p*	Beta	*t*	*p*	Beta	*t*	*p*	Beta	*t*	*p*
Time 2 Anhedonia	0.20	0.13	0.12	4.11	**< 0.001**	− 0.08	-1.35	0.18	− 0.14	-2.41	**0.02**	0.31	17.31	**<. 001**
Time 2 Asociality	0.25	0.11	0.06	1.88	0.06	− 0.06	− 0.85	0.40	− 0.13	-2.10	**0.04**	0.30	16.88	**< 0.001**
Time 2 Avolition	0.19	0.07	0.09	2.68	**0.007**	− 0.09	-1.19	0.23	− 0.14	-2.20	**0.03**	0.23	13.08	**< 0.001**
Time 2 Depressed mood	0.39	0.25	0.18	6.20	**< 0.001**	− 0.33	-5.02	**< 0.001**	− 0.10	-1.62	0.11	0.30	16.72	**< 0.001**

Standardized betas are reported. Note that the fit was singular for the AR avolition model, so we removed nested within day to fit the model. Significant p-values (p < .05) are bolded for ease of interpretation.

*DPB*  = defeatist performance beliefs

### DPB time and context effects

Mixed-effects models by group examining time and context effects accounted for approximately 90% of the variance in DPB scores, indicating that the majority of variance is due to between-person rather than with within-person factors. However, time and context variables did demonstrate effects above and beyond group alone.

### Time effects

*Day of EMA data collection period*. For day of EMA, there was a significant main effect of group and day but not an interaction between group and day (see Table 4). The day effect was such that DPB were significantly higher on the first day compared to any other study day. No other significant differences were observed between the study days.

*Day of the week.* For day of the week, there was also a significant main effect of group and weekday. DPB were higher in the middle of the week (Wednesday) compared to Saturday, Sunday, and Monday. DPB on Thursday were also significantly higher than DPB on Sundays.

*Time of Day*. For time of day, there was a significant group and time of day effect. The group X time of day effect was trending (*p* = .06). Post-hocs demonstrated that DPB were significantly higher in the morning than the afternoon or evening. No significant differences were observed between the afternoon and evening.

### Context effects

Activity. For activity, main effects of Group and Activity Context were significant (See Table 4), while the Group X Context effect was non-significant. Across both groups, DPB were significantly elevated (compared to recreation activities) when working, eating, computer, resting, and running errands. Thus, when engaged in goal-directed or more passive activities, DPB were more elevated than during recreation activities.

Location. There was a significant main effect of group but not of location context or a group X location context interaction. This suggests that across the locations of home, work, public, house of family, on average DPB did not significantly differ.

Social partner. For social partner, these was a significant group main effect but not a significant main effect for social partner. However, there was significant interaction between social partner and group. Post-hocs indicated that CHR had greater DPB around strangers than significant others and friends, while DPB levels did not vary across social partners for healthy controls.

Social modality. There was a significant main effect of group but not of social modality or a group X social modality interaction. This suggests that across the modalities of social interaction (in person, electronic, phone), on average, DPB did not significantly differ.

**Table 4:** Omnibus model effects for DPB across contexts

Predictor	Total *R*^2^	Fixed-effect *R*^2^	Group	Predictor	Group X Predictor
*Day of EMA, week, time of day*					
Day of EMA	0.90	0.10	21.94 ***	30.59***	4.38
Day of week	0.90	0.10	16.81***	21.93**	9.75
Time of day	0.90	0.10	20.49***	16.41***	5.69
*Context*					
Activity	0.91	0.10	19.29***	17.93**	3.97
Location	0.91	0.10	21.10***	3.17	1.51
Social partner	0.91	0.09	22.31***	7.28	9.97*
Social modality	0.90	0.10	22.35**	3.22	3.18

*p < 0.05

**p < 0.01,

***p < 0.001

## Discussion

Identification of temporally precise mechanisms that contribute to real-time increases in reduced goal-directed behavior, experience of pleasure, and social drive in CHR youth could yield more effective negative symptom treatment approaches, including informing on the utility of dynamic, real-time mHealth treatments. Guided by CBT models of negative symptoms [35–37], the primary aim of this study was to examine whether a specific type of biased thinking—DPB—was a real-world mechanism contributing to negative symptoms in CHR youth. A secondary aim was to identify whether DPB is also a dual mechanism of another common symptom of CHR: depressed mood. To lay the ground-work for identifying whether mHealth interventions may be useful for targeting DPB in daily life, we also conducted analyses to determine when and how DPB fluctuates across time of day, study day, and environmental contexts (i.e., to identify if there are central times when DPB should be targeted).

### Do CHR report elevated DPB compared to CN?

This study represents the first attempt to our knowledge to examine DPB in CHR youth in real-world environments outside of the laboratory. Results demonstrated that CHR youth report elevated DPB in their daily lives compared to CN. These results are consistent with the CBT model of negative symptoms [35, 37] suggesting that these beliefs develop prior to the onset of a first psychotic episode in part through repeated exposure to discouraging life experiences. These findings therefore provide real-world evidence that these beliefs do not develop only in response to a schizophrenia diagnosis, the associated stigma, or medication effects. Further, results extend prior laboratory-based studies identifying that DPB are elevated in CHR youth on the trait DPB scale [12, 22, 23]. Our results suggest that these findings have external validity and are not due to recall biases or other biases that can impact accurate reporting on trait self-report scales. Thus, even as CHR youth go about their daily lives, they experience elevations in negative perceptions about potential failure, which in turn increases depressed mood and reduces engagement in goal-directed and pleasurable activities.

## Are greater DPBs associated with greater concurrent negative symptoms and depressed mood measured via EMA?

Our results also identify DPB as an important potential transdiagnostic mechanism in CHR youth. Given that mood abnormalities are frequently present in CHR youth, and CHR youth frequently go on to develop mood rather than psychotic disorders [27–29], identifying mechanisms that contribute to both symptoms of psychotic and mood disorders may be critical for treatment planning. We observed that elevations in DPB predicted both concurrent elevations in all domains of negative symptoms (avolition, anhedonia, asociality) as well as increased depression across both CHR youth and CN. This real-world test of CBT models of depression and negative symptoms provides evidence that these DPB elevations are associated with multiple symptoms in the daily lives of CHR youth. These findings extend prior laboratory-based studies which observed associations between greater trait DPB and depression symptoms in CHR youth [22]. Targeting DPB may therefore optimize prevention efforts and outcomes in the largest group of CHR youth, given that DPB is a potential shared mechanism of symptoms core to the disorders CHR youth most frequently develop: psychotic and mood disorders [28].

### Do DPB at time t predict the change in negative symptoms and depressed mood from time t to t + 1?

This study also provides one of the strongest tests of how a promising transdiagnostic mechanism, DPB, can impact subsequent increases in both negative symptoms and depressed mood. Results supported the hypothesized mechanistic CBT pathway of increases in DPB leading to subsequent elevations in negative symptoms and depressed mood by indicating that increased DPB at time *t* was associated with greater negative symptoms for all domains (anhedonia, avolition, and asociality) and depressed mood at *t* + 1 after controlling for the baseline symptom at time *t.* There was also a significant Group X DPB interaction for all three negative symptom domains but not depressed mood, with post-hoc analyses demonstrating a stronger positive association between DPB and negative symptoms in CHR than CN. This suggests the detrimental effect of DPB on subsequent negative symptoms is stronger in CHR than CN, while the effect of DPB on prospective depressed mood is similar across CHR and CN. This provides specificity to the CBT model of negative symptoms, suggesting that DPB may be an especially potent target for reducing negative symptoms in the daily lives of CHR youth. On the other hand, as would be expected as depression (versus a psychotic disorder diagnosis) could more likely develop in both CHR and CN, our results offer less specificity for the role of DPB on depressed mood in CHR youth and instead suggest that targeting DPB in CBT treatments could be equally beneficial across CHR youth and CN. This may suggest that elevated DPB may be more of a unique risk factor for negative symptoms in CHR youth, and across different clinical and non-clinical groups, elevated DPB may also be a real-world contributor to depressed mood. Targeting DPB in CHR youth may therefore help to reduce negative symptoms as well as depressed mood.

Our study is also the first to provide a proof-of-concept evaluation for whether mHealth treatments may be promising approaches for targeting DPB in CHR youth. Beyond demonstrating a lagged association between negative symptoms and depressed mood in CHR youth, our analyses also identified key moments where mHealth treatments may be most useful for targeting DPB. Results identified that DPB fluctuated across (1) time of day, (2) day of the week, (3) study day, and (4) environmental contexts. On average, DPB were highest in the morning, middle of the week, and first day of data collection. DPB also varied across contexts, with DPB severity significantly varying across activities and type of social partner but not location or method of social interaction. For activities, DPB were elevated in both groups when participants were engaging in goal-directed *or* more passive activities compared to recreational activities. Recreational activities – or activities that might help to activate more positive beliefs (e.g., I am able to do this; I am able to get enjoyment from this) [38] – may therefore help to mitigate DPB, while DPB might be more elevated when working on challenging activities or when participants are engaging in avoidance behaviors (passive activities) in response to DPB. In the CHR group alone, DPB were greater when CHR were around strangers compared to significant others and friends. Thus, these may be key contexts where DPB interventions may be most effective. However, notably the large R^2^ values in these time and context analyses also indicated that a large portion of the variance is at the person-level.

These findings together suggest that a combination of static, pre-scheduled, and just-in-time mHealth approaches [39] might be needed to target DPB in the daily lives of CHR. For example, intervention content may be most beneficial in the morning and during the middle of the week on average. In addition, to address the individual variability in DPB elevations, intervention content could be automatically triggered based on elevations in reported DPB. This could be done by administering brief EMA surveys containing these DPB items at randomized times throughout the day. Then, exactly when a person might need and could benefit the most from a CBT skill to help counter elevations in DPB, intervention content could be automatically delivered to their phone. In combination with the negative symptom EMA items, examination of DPB levels and subsequent negative symptoms measured via EMA after the provision of intervention content could provide a real-world mechanism-engagement test and afford examination of how engagement in the targeted DPB mechanisms yields changes in negative symptoms. Overall, a mHealth treatment approach could reduce the subsequent increase observed in both negative and depressive symptoms and help CHR youth implement more skills in their daily lives that could help to lessen the severity of future DPB. This is a promising approach to help offset the development of more severe depressed mood and negative symptoms, which could potentially reduce the likelihood of the development of a formal disorder.

### Limitations

Certain limitations should be considered when interpreting these findings. First, EMA data was collected across one week; it is possible that different patterns could emerge over longer periods; longitudinal studies could help to clarify whether the effects differ based on those who transition to a psychotic disorder or a mood disorder. Second, our findings only help to inform mHealth treatment feasibility and utility, and future work is needed to identify how to best intervene on these beliefs in daily life and how targeting these beliefs could influence subsequent symptoms. Finally, our EMA measure of depressed mood did not capture the full range of symptoms important for depression.

## Conclusions

This study provides real-world support for CBT models of psychopathology in individuals at CHR. Findings demonstrate that DPB are elevated in CHR youth in their daily lives and linked to both increased concurrent and prospective depressed mood and negative symptoms in CHR youth. These results provide one of the strongest mechanistic tests of the CBT models of psychopathology in CHR youth to date, identifying how beliefs about potential failure contribute to dynamic changes in subsequent negative symptoms and depressed mood only 15 to 90 min later. In other words, these findings also suggest that even prior to the development of a full psychotic disorder, DPB are a potent, real-world mechanism underlying negative symptoms and depressed mood. Digital mHealth-based treatment approaches may be useful for targeting DPB using static, pre-scheduled, and just-in-time approaches Together, this could provide youth therapeutic support in their daily lives and in some of the exact moments when they might need help the most, potentially offsetting the development of more severe symptoms.

## Supplementary Information

Below is the link to the electronic supplementary material.


Supplementary Material 1

