# Supplementary material for: Elevated defeatist performance beliefs predict state increases in negative symptoms in daily life in clinical high-risk for psychosis youth: implications for mobile health treatments
Source: Eur Arch Psychiatry Clin Neurosci. 2025 Nov 10;276(6):2811–20. doi: 10.1007/s00406-025-02143-4 (PMC13526039; doi:10.1007/s00406-025-02143-4)
Supplement: Supplementary file 1 [file 406_2025_2143_MOESM1_ESM.docx]

**Supplemental Materials for**

**Elevated Defeatist Performance Beliefs Predict State Increases in Negative Symptoms in Daily Life in Clinical High-Risk for Psychosis Youth: Implications for Mobile Health Treatments**

Lauren Luther, Ph.D.^1^

Ian M. Raugh, Ph.D.^2^

Luca Bela G. Webber^1^

Paul M. Grant, Ph.D.^3^

Aaron T. Beck, M.D.^3,4^

Vijay A. Mittal, Ph.D.^5,6^

Elaine Walker, Ph.D.^7,8^

Gregory P. Strauss, Ph.D.^9^

1. Department of Psychology, University of Alabama at Birmingham, Birmingham, AL, USA
2. Douglas Mental Health Institute, Department of Psychiatry, McGill University, Montréal, Québec, CA
3. Center for Recovery-Oriented Cognitive Therapy, Beck Institute, Bala Cynwyd, PA, USA
4. Department of Psychiatry, University of Pennsylvania, Philadelphia, PA, USA
5. Department of Psychology, Northwestern University, Evanston, IL, USA
6. Institutes for Policy Research (IPR) and Innovations in Developmental Sciences (DevSci), Psychiatry, Medical Social Sciences, Northwestern University,  Evanston, IL, USA
7. Department of Psychiatry and Behavioral Sciences, Emory University, Atlanta, GA, UGA
8. Department of Psychology, Emory University, Atlanta, GA, UGA
9. Department of Psychology, University of Georgia, Athens, GA, UGA

*Correspondence concerning this article should be addressed to Gregory P. Strauss, Ph.D., Email: gstrauss@uga.edu. Phone: +1-706-542-0307. Fax: +1-706-542-3275. University of Georgia, Department of Psychology, 125 Baldwin St., Athens, GA 30602.

***EMA Methods***

Eight daily surveys were sent across the six-day EMA period.

*Negative Symptoms.* Each daily survey contained negative symptom items, which are listed below. To minimize burden, skip logic was used so that only follow-up questions were asked if a relevant behavior was endorsed. Participants were instructed to fill out each daily survey based on the past 15 minutes. For questions with multiple options (see below), participants were instructed to indicate as many options that were applicable in this time frame.

1.1 What are you doing?^M^ [**Used to assess behavioral component of avolition and anhedonia; engagement in any of the noted behaviors below was scored as 1 for behavioral component]**
    1.1.1 Working/ Studying **[avolition behavioral component]**
    1.1.2 Eating/ Drinking
    1.1.3 Recreation/ Hobby **[anhedonia behavioral component]**
    1.1.4 Internet/ Computer use
    1.1.5 Errands/ Housework **[avolition behavioral component]**
    1.1.6 Resting
    1.1.7 Exercising **[avolition behavioral component]**
    1.1.8 Shopping **[avolition behavioral component]**
    1.1.9 TV/ Music
    1.1.10 Smoking
    1.1.11 Commuting/ Traveling **[avolition behavioral component]**
    1.1.12 Bathing/ Hygiene
    1.1.13 Pacing restlessly
    1.1.14 Socializing
    1.1.15 Nothing *(Proceed to 1.3)*
1.2 Please complete the following:^C^
    1.2.1 How interested are you in the activity? **[Avolition internal experience item]**
    1.2.2 How much are you enjoying the activity? **[Anhedonia – consummatory internal experience item]**
    1.2.3 How much do you think you will enjoy that activity the next time you do it? **[Anhedonia – anticipatory internal experience item]**
1.3 *(If 1.1.15 selected)* How much do you want to be doing an activity right now?^C^
1.4 Who are you interacting with?^M^ **[Asociality behavioral item]**
    1.4.1 Significant other
    1.4.2 Family/ Roommates
    1.4.3 Friends
    1.4.4 Coworkers/ Classmates
    1.4.5 Doctor/ Therapist
    1.4.6 Strangers
    1.4.7 No one/ Alone *(Proceed to 1.6)*
1.5 Please complete the following:^C^
    1.5.1 How interested are you in this social interaction? **[Asociality internal experience Item]**
    1.5.2 How much are you enjoying this social interaction? **[Anhedonia – consummatory internal experience item]**
    1.5.3 How much do you think you will enjoy interacting with them next time? **[Anhedonia – anticipatory internal experience item]**
1.6 *(If 1.4.7 selected)* How much do you want to be interacting with someone right now?^C^

Answer format for EMA questions is indicated as follows by the superscripts after the question text:

C = Continuous slider from 0 on left to 100 on right

M = Multiple selection among sub-options

Scoring for each negative symptom domain via was calculated as engagement in the behavioral component (scored 0 or 1) +1) X internal experience item (scored 0-100). More specifically:	(behavioral component + 1) * internal component = domain score.

**Defeatist Performance Beliefs EMA Items.** Each daily survey contained Defeatist Performance Belief items, which are listed below.

1.1	How much do you agree or disagree with the following right now:^C^

1.1.1 I have to do well all the time or people will not respect me.
1.1.2 If you cannot do something well, there is little point in doing it.
1.1.3 If I fail at all, it is as bad as being a complete failure.

C = Continuous slider from 0 on left to 100 on right

**Depressed Mood EMA Items.** Each daily survey contained a depressed mood item, which is listed below.

1.1 How _____ do you feel right now?^C^
    1.1.1 Sad, downhearted, unhappy

C = Continuous slider from 0 on left to 100 on right
